# Infant Colic Symptoms and Amino Acid Formula: Insights from a Prospective Cohort Study

**DOI:** 10.3390/nu17081302

**Published:** 2025-04-09

**Authors:** Jerry M. Brown, Jessica V. Baran, Luke Lamos, Jesse Beacker, Jared Florio, Lea V. Oliveros, Abigail L. Fabbrini, Andrew A. Farrar, Michael J. Wilsey

**Affiliations:** 1 Office of Medical Education, Florida Atlantic University Charles E. Schmidt College of Medicine, Boca Raton, FL 33431, USA; jerrybrown2020@health.fau.edu (J.M.B.); jessicavbaran@gmail.com (J.V.B.); llamos2020@health.fau.edu (L.L.); jbeacker2021@health.fau.edu (J.B.); jflorio2014@health.fau.edu (J.F.); 2 Office of Medical Education, Alabama College of Osteopathic Medicine, Dothan, AL 36303, USA; oliverosl8517@acom.edu; 3 Office of Medical Education, Kansas City University College of Osteopathic Medicine, Kansas City, MO 64106, USA; abby.fabbrini@yahoo.com (A.L.F.); farrardrew@gmail.com (A.A.F.); 4 Department of Pediatrics, University of South Florida Morsani College of Medicine, Tampa, FL 33606, USA

**Keywords:** infant colic, cow’s milk protein allergy, infant nutrition, amino acid formula, ZSMoments, mobile health application

## Abstract

**Background/Objectives:** Infant functional disorders, including colic, irritability, and sleep disturbances, often overlap with symptoms of cow’s milk protein allergy (CMPA). While extensively hydrolyzed formulas are commonly used to address these issues, the short-term efficacy of amino acid formulas (AAF) for managing colic remains understudied. This secondary analysis of a previously published prospective cohort, the largest of its kind in the United States, evaluated the short-term impact of AAF in improving colic and associated symptoms in infants under six months of age with suspected CMPA. **Methods:** This real-world prospective cohort study analyzed data from 138 infants with suspected CMPA initiated on AAF at Visit 1. After excluding 34 infants due to incomplete data or treatment changes, 104 infants were included in the final analysis. Symptom severity and resolution were assessed through outcomes across two visits, with care provided by general pediatricians (82%) and pediatric gastroenterologists (18%). **Results:** At baseline, the most common symptoms were colic (*n =* 83), gassiness (*n =* 72), fussiness (*n =* 66), and sleep disturbances (*n =* 58). By the next follow-up visit (Visit 2), parents reported symptom improvements in colic (94%), gassiness (81%), fussiness (83%), and sleep disturbances (86%). Complete symptom resolution was observed in 73% of infants with colic, 68% with gassiness, 65% with fussiness, and 81% with sleep difficulties. By Visit 2, no infants had severe symptoms, with only mild residual symptoms reported. **Conclusions:** AAF was associated with significant short-term improvements in colic and related symptoms in infants with suspected CMPA. These preliminary findings highlight AAF as a promising dietary intervention during early infancy. Larger controlled studies are necessary to confirm these results and explore long-term outcomes.

## 1. Introduction

An estimated 20% to 30% of infants experience infant colic within the first 6 months of life, and though it is relatively common, it is a generally self-limiting condition [1]. Infants with colic often exhibit prolonged periods of inconsolable crying, along with raising of the legs, clenching of fists, and facial flushing [2]. Despite the prevalence of this condition, researchers have yet to determine its pathogenesis, and there is little consensus on its underlying mechanisms or overall etiology [3,4,5,6]. Clinicians rely on Rome IV criteria for diagnosing infant colic, which requires infants to be < 5 months old at onset, show no signs of failure to thrive, fever, or illness, and have unexplained recurrent periods of crying that caregivers cannot abate [3]. Symptoms of colic, including crying, irritability, flatulence, and sleeping difficulties, can lead to anxiety and depression in parents and have been linked to instances of child abuse [7,8,9,10]. According to the National Health Service, the healthcare system bears a significant cost of USD 104 million annually for treating infant colic [11]. These emotional and substantial economic costs underscore the need for an improved understanding of the pathogenesis of this condition and for healthcare providers to have better colic management strategies [12].

Treatment options for infant colic are limited, with most showing only moderate evidence of effectiveness [13]. Certain probiotics have demonstrated short-term efficacy in reducing colic symptoms [14]. Functional disorders in infancy, such as colic, irritability, and sleep disturbances, are often associated with cow’s milk protein allergy (CMPA). This prevalent food allergy shares symptoms with colic, including crying, fussiness, flatulence, and sleep difficulties [8,15].

Evidence suggests that eliminating certain allergenic foods (e.g., cow’s milk, eggs, fish, peanuts, soy, tree nuts, wheat) from breastfeeding mothers’ diets may reduce colic symptoms [7,16]. In formula-fed infants, first-line treatment typically involves extensively hydrolyzed formula (eHF), with amino acid formulas recommended for severe CMPA or cases unresponsive to eHF. While extensively hydrolyzed formulas have shown promise in alleviating symptoms like crying and irritability [17,18], there is a lack of studies examining the short-term use of amino acid formulas for managing infant colic. Additionally, the importance of achieving short-term symptom relief cannot be overstated, as it is a priority for parents and pediatric physicians seeking to quickly restore infant health and well-being [19,20,21].

This secondary analysis of a prospectively collected cohort aimed to evaluate changes in colic symptom severity in symptomatic infants under 6 months of age managed with a commercial amino acid formula (AAF) in infants with suspected CMPA. The primary objective of this real-world study was to assess short-term improvements in colic and related symptoms after initiating an AAF as prescribed by general pediatricians and pediatric gastroenterologists. We hypothesized that colic and associated symptoms would decrease in severity by the next follow-up visit (Visit 2) compared to the initial clinical diagnosis and enrollment (Visit 1).

## 2. Materials and Methods

### 2.1. Study Population

This study is a secondary analysis of a prospective cohort in the United States, the largest of its kind, evaluating the short-term symptom improvement of AAF in symptomatic infants under six months of age with suspected CMPA in a real-world clinical setting [22,23]. Pediatric HCPs evaluated infants included in the analysis for colic symptoms, gassiness, fussiness, and sleep difficulties from July 2021 to October 2021. They were prescribed a commercially available amino acid formula (PurAmino™, Mead Johnson Nutrition, Evansville, Indiana) as clinically indicated at Visit 1. They continued the formula use through the next outpatient follow-up visit (Visit 2). Infants were excluded from the analysis if they did not receive AAF at Visit 1, were over six months old at the time of formula initiation, had incomplete survey data at Visits 1 or 2, or discontinued or switched formulas between visits. This observational cohort study was approved by the Johns Hopkins All Children’s Institutional Review Board (IRB00279920) on 28 April 2021.

### 2.2. Data Collection

As previously described, all patient data were de-identified at Visit 1 by assigning each patient a unique animal name identifier, ensuring no personally identifiable information (e.g., name or birth date) was recorded [22,23]. Pediatric HCPs documented demographic information and baseline symptoms and assigned a study formula. Data were collected using ZSMoments™, a secure, mobile-based platform developed by ZS Associates (Bellevue, WA, USA). This mobile health application, installed on the HCP’s mobile device, enabled real-time data capture in outpatient clinical settings through an efficient interface that facilitated seamless data entry and patient de-identification, ensuring compliance with privacy standards.

For this secondary analysis, symptoms of interest included colic, gassiness, fussiness, and sleeping difficulties. Symptom severity was rated on a 0–3 scale: 0 (Not Present), 1 (Low), 2 (Moderate), 3 (Severe), or Not Assessed. An aggregate symptom score, combining individual symptom ratings, was calculated at both visits to assess changes in severity over the short term. Improvement was defined as a decrease in the aggregate symptom score from Visit 1 to Visit 2, while “no improvement” indicated no change in scores (e.g., an aggregate score of 4 at both visits). Sub-cohort analyses focused on patients presenting with specific symptoms at Visit 1 to evaluate symptom improvement and resolution. No post hoc analyses were performed.

### 2.3. Statistical Analyses

Continuous study variables were summarized with medians and ranges (minimum/maximum), and categorical study variables were reported as counts and percentages [24]. Data were inspected for completeness and outliers before analyses, and a complete case analysis was used to analyze variables with missing data. Statistical significance was evaluated using *t*-tests to calculate *p*-values between proportions, with alpha < 0.05 considered significant. All analyses were conducted with an SPSS-based analytics tool (IBM, Armonk, NY, USA).

## 3. Results

**Figure 1:**
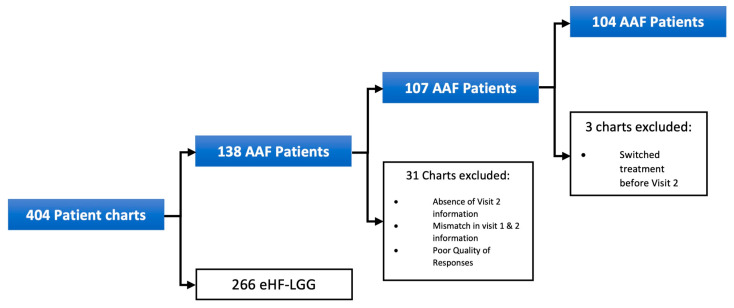
Patient exclusion for initial AAF cohort.

Pediatric HCPs started 138 symptomatic infants with clinically suspected CMPA on AAF at Visit 1. However, 31 patients were excluded from the final sample due to incomplete data. Among the 107 patients in the final sample, 88 (82%) were managed by general pediatricians, while 19 (18%) were seen by pediatric gastroenterologists. Three additional patients were excluded from the symptom analysis due to treatment switching before Visit 2. Further exclusions for patients not assessed for specific infant colic symptoms are detailed in Figure 1.

**Figure 2:**
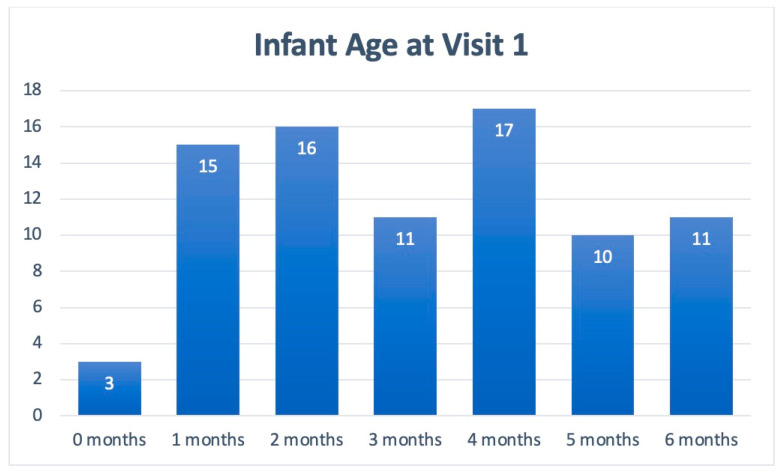
Age stratification of patients presenting with infant colic symptoms at Visit 1.

Table 1 includes patient demographics collected from Visits 1 and 2, while Figure 2 depicts the cohort’s age stratification. Of the patients included in the symptom analysis, 75% were under 5 months of age, and the majority were male (60%; *n =* 64). Across visits, the symptoms of all 104 infants in the cohort were assessed. Due to the natural progression of each clinical course, some patients presented with new symptoms at Visit 2.

As shown in Table 2, most symptomatic infants with colic and related symptoms experienced improvement or resolution of symptoms at Visit 2. Additional analyses focused on infants presenting specific symptoms at Visit 1, including colic (*n =* 83), gassiness (*n =* 72), fussiness (*n =* 66), and sleep difficulties (*n =* 58). By Visit 2, 94% of infants with colic and 81% with gassiness improved, 86% experienced improved sleep, and 83% reported reduced fussiness.

Parents of the 83 symptomatic infants with infant colic at Visit 1 reported that 61 (73%) experienced complete resolution of symptoms by Visit 2. Similarly, among the 66 infants with fussiness, 46 (70%) were reported to have total resolution of their symptoms. Complete resolution was also reported for 50 of the 72 infants with gassiness (69%) and 48 of the 58 infants with sleep difficulties (83%). Table 3 provides further details on symptom resolution by initial severity. For instance, among the 18 infants presenting with severe colic at Visit 1, 14 (78%) were reported to have complete resolution by Visit 2.

This analysis showed that amino acid formula-fed infants experienced a significant reduction in reported colic and related symptom severity between Visit 1 and Visit 2. Most patients showed statistically significant symptom improvement, with nearly all symptomatic infants at Visit 2 presenting with only mild symptoms. Notably, no infants exhibited severe symptoms at Visit 2.

## 4. Discussion

This real-world prospective cohort study analyzed data from 104 symptomatic infants who were started on an amino acid formula (AAF) at their initial clinical presentation (Visit 1) and followed up at the next outpatient visit (Visit 2). Symptom severity and resolution were evaluated through outcomes across these two visits, with care provided by general pediatricians (82%) and pediatric gastroenterologists (18%).

This secondary analysis focused on colic and associated symptoms and found that the vast majority of infants experienced improvements across all symptoms assessed by clinicians at Visit 2. Notably, no infants had severe symptoms at follow-up, with only mild residual symptoms remaining. Additionally, the majority of patients who presented with a given symptom at Visit 1 experienced total resolution of that symptom at Visit 2 as seen in Figure 2. These findings highlight the potential of AAF to provide rapid relief for infant colic-related symptoms—often by the next follow-up visit—addressing the need to develop effective short-term colic management strategies [1,2,3,4,25,26].

While extensively hydrolyzed formulas are commonly used to address CMPA-related symptoms and, more recently, infant colic symptoms [20], the short-term efficacy of AAF for managing colic in infants remains understudied [26]. One previous six-patient case series observed exclusively breastfed infants and measured baseline crying/fussing time over 3–5 days before transitioning to an amino acid formula for 4–8 days [26]. Infants experienced significantly reduced crying times within 1 day of AAF initiation. This secondary analysis expands on the original cohort study—the largest of its kind in the United States—by focusing on the short-term impact of AAF on colic and numerous associated symptoms in infants during the first six months of life [22].

Infant colic is a common yet poorly understood condition characterized by episodes of excessive, inconsolable crying in an otherwise healthy infant [1,2,3,4,5,6]. It typically begins in the first few weeks of life, peaks around six weeks, and often resolves naturally by six months of age, with prevalence rates ranging from 10% to 40% [6,7,8,9,10]. While crying is a normal form of communication for infants, colic is distinguished by facial flushing, fist clenching, and prolonged, intense episodes of crying that are resistant to soothing efforts [2,3,4].

The etiology of colic remains elusive, with proposed mechanisms including increased intraluminal gas, gut dysmotility, visceral pain, food sensitivities, and environmental or psychosocial factors [5,10]. However, evidence supporting gastrointestinal dysfunction as the primary cause is inconclusive. Some studies, such as those by De Weerth et al., suggest that gut dysbiosis—reduced microbiota diversity and stability—may play a role in infant colic [27].

Though colic is generally self-limiting, its unpredictable, disruptive, and unexplained nature places significant emotional and physical strain on caregivers [1,2,3,4,5]. Feelings of inadequacy, anxiety, and depression are common among parents, compounding the challenges of managing the condition and prompting frequent healthcare visits [6,7,8,9,10,11].

Management strategies for colic remain varied, with limited robust evidence supporting specific interventions [1,2,3,4,25]. Recent research highlights the potential benefits of probiotics, such as *Lactobacillus rhamnosus GG* (LGG), shown in prospective controlled trials to reduce crying time and fecal calprotectin levels in infants with colic [28]. Similarly, *Lactobacillus reuteri* and certain *Bifidobacterium animalis* subspecies have helped reduce crying and fussing time in exclusively breastfed infants [29,30,31,32,33]. A meta-analysis of randomized controlled trials found that *L. reuteri* nearly doubled the likelihood of treatment success compared to placebo in breastfed infants [14]. However, its benefits in formula-fed infants remain uncertain.

For formula-fed infants with CMPA and symptoms resembling colic, extensively hydrolyzed formulas, including those containing LGG, are often first-line treatments. In severe cases or when extensively hydrolyzed formulas are not tolerated, amino acid formulas (AAF) may be used. However, there is a notable gap in the literature regarding the efficacy of AAF in managing colic symptoms [26]. Despite this limitation, AAF remains an important option for infants with co-occurring CMPA, highlighting the need for further research to better understand its role in colic management [25,26].

Despite ongoing research, the pathogenesis of colic remains unclear, necessitating further exploration of its underlying mechanisms and the effectiveness of current management strategies [12]. Addressing this knowledge gap is essential for alleviating the burden of colic on infants and their families [6,7,8,9,10,11].

When considering the management of infant colic with amino acid formula (AAF), it is important to weigh its clinical benefits against its higher cost compared to standard formulas. While AAF has shown promise as a potential management strategy [23], the financial impact on families and healthcare systems raises the need for a thorough cost–benefit analysis. Future research should focus on comparing these costs to help guide informed decisions about using AAF in managing colic.

We acknowledge several limitations in our study. Voluntary clinician participation may have introduced selection and response biases, and the focus on the U.S. may limit generalizability to other patient populations. Reliance on clinician assessment and judgment of infant colic, without strict adherence to Rome IV criteria, may have affected case identification. The real-world study design and absence of a control group limits comparisons to other treatments or no treatment. Including symptomatic infants over 4 months at the time of presentation raises the possibility of spontaneous symptom resolution before the next follow-up visit [34,35]. Despite efforts to standardize data collection, the natural progression of colic and potential placebo effects cannot be ruled out. Future research should include control groups, assessing long-term outcomes, and addressing these limitations.

## 5. Conclusions

This study highlights the potential of AAF to provide short-term improvement in colic symptoms, such as gassiness, fussiness, and sleep difficulties, in symptomatic infants. By addressing symptoms, our findings underscore the importance of short-term relief—a priority for both parents and pediatric providers. As the largest prospective study in this area, this secondary analysis helps address the knowledge gap in exploring the role of AAF in managing colic, particularly in infants with suspected CMPA. While these initial results may suggest potential benefits, further controlled studies are needed to confirm these preliminary results and to explore the broader implications of AAF use in this context.

## Figures and Tables

**Table 1:** Patient demographics.

Item	Population
Sex	
Male, n (%)	64 (60)
Female, n (%)	43 (40)
Mean age at enrollment, months (Range)	3 (1–6)
Median time between visits, weeks (IQR)	5 (4–6)

IQR: Interquartile range.

**Table 2:** Symptom burden of the cohort at each visit.

	Visit 1					Visit 2				
Symptom	*n*	Severe	Moderate	Mild	Not Present	*n*	Severe	Moderate	Mild	Not Present
Colic	104	17%	36%	27%	20%	103	0%	2%	19%	79%
Fussiness	101	7%	26%	30%	38%	101	0%	1%	19%	80%
Gassiness	101	10%	17%	45%	29%	102	0%	3%	20%	77%
Sleep Difficulties	100	7%	21%	28%	44%	101	0%	1%	9%	90%

**Table 3:** Rate of total symptom resolution at first follow-up visit by severity at initial presentation.

Symptom	Severe in Visit 1 - > Total Resolution in Visit 2	Moderate in Visit 1 - > Total Resolution in Visit 2	Mild in Visit 1 - > Total Resolution in Visit 2
Colic	14 (78%)	24 (65%)	23 (82%)
Fussiness	4 (57%)	20 (77%)	22 (73%)
Gassiness	6 (60%)	12 (71%)	32 (71%)
Sleeping Difficulties	7 (100%)	19 (90%)	22 (79%)

## Data Availability

The datasets generated and/or analyzed during the current study are not publicly available due to subject confidentiality, but are available from the corresponding author on reasonable request.

## Abbreviations

The following abbreviations are used in this manuscript:

CMPA	Cow’s milk protein allergy
eHF	Extensively hydrolyzed formula
AAF	Amino acid formula
HCP	Healthcare provider
GI	Gastroenterology
LGG	*Lactobacillus rhamnosus* GG
